# Microcomputed tomography of the femur of diabetic rats: alterations of trabecular and cortical bone microarchitecture and vasculature—a feasibility study

**DOI:** 10.1186/s41747-019-0094-5

**Published:** 2019-04-11

**Authors:** David Zeitoun, Guavri Caliaperoumal, Morad Bensidhoum, Jean Marc Constans, Fani Anagnostou, Valérie Bousson

**Affiliations:** 1 Centre hospitalier Lariboisière, Hopital Lariboisière, Service de radiologie ostéo-articulaire, 2 rue Ambroise Paré, 75010 Paris, France; 2 CNRS Laboratoire B2OA, Laboratoire B2OA.10, Avenue de Verdun, 75010 Paris, France; 3 Centre hospitalier Amiens, Chu Amiens, Service de radiologie, Chemin de Longpré, 80080 Amiens, France

**Keywords:** Bone and bones, Diabetes mellitus (type 2), Rats (Zucker), Vascular diseases, X-ray microtomography

## Abstract

**Background:**

To better understand bone fragility in type 2 diabetes mellitus and define the contribution of microcomputed tomography (micro-CT) to the evaluation of bone microarchitecture and vascularisation, we conducted an *in vitro* preliminary study on the femur of Zucker diabetic fatty (ZDF) rats and Zucker lean (ZL) rats. We first analysed bone microarchitecture, then determined whether micro-CT allowed to explore bone vascularisation, and finally looked for a link between these parameters.

**Methods:**

Eight ZDF and six ZL rats were examined for bone microarchitecture (group 1), and six ZDF and six ZL rats were studied for bone vascularisation after Microfil® perfusion which is a radiopaque casting agent (group 2). In group 1, we used micro-CT to examine the trabecular and cortical bone microarchitecture of the femoral head, neck, shaft, and distal metaphysis. In group 2, micro-CT was used to study the blood vessels in the head, neck, and distal metaphysis.

**Results:**

Compared to ZL rats, the ZDF rats exhibited significantly lower trabecular bone volume and number and higher trabecular separation in the three locations (*p* = 0.02, *p* = 0.02, *p* = 0.003). Cortical porosity was significantly higher in the ZDF rats at the neck and shaft (*p* = 0.001 and *p* = 0.005). We observed a dramatically poorer bone vascularisation in the femur of ZDF rats, especially in distal metaphysis (*p* < 0.047).

**Conclusions:**

Micro-CT demonstrated not only significant alterations in the bone microarchitecture of the femurs of ZDF rats, but also significant alterations in bone vascularisation. Further studies are required to demonstrate the causal link between poor vascularisation and impaired bone architecture.

## Key points

- Microcomputed tomography (micro-CT) is a promising tool to study bone vascularisation
- Trabecular and cortical bone quantity and microarchitecture were altered in the femur of Zucker diabetic fatty (ZDF) rats
- In ZDF rats, an impaired bone architecture is associated to a poor bone vascularisation
- The main region of interest for studying architecture and vasculature changes in the femur of ZDF rats is the distal metaphysis

## Background

Type 2 diabetes mellitus (T2DM) is a disease whose worldwide incidence is increasing [1]. Morbidity and mortality of this disease are mainly due to vascular complications, but more recently, increased risk of fractures has emerged as a serious complication in patients with long-standing or poorly controlled disease [2, 3]. This increased risk of fractures has a distinct propensity for the proximal end of the femur [4–7] and vertebrae [8] and is problematic because patients with T2DM exhibit compromised bone fracture healing and poorer outcomes after fracture [9–11].

A paradox of this clinical syndrome is that increased risk of bone fracture is evident despite higher body weight and normal-to-high areal bone mineral density [12], which are generally associated with reduced bone fracture risk. The increased risk of bone fracture is also not explained by a higher frequency of falls among T2DM patients [13]. Several studies using high-resolution peripheral quantitative computed tomography in T2DM patients found that the microarchitecture of the trabecular bone tissue had been preserved but cortical porosity had increased and bone quality was impaired [14–16]. Definitive elucidation of the pathogenesis of these abnormalities will require further studies.

The use of animal models is essential in research pertaining to T2DM. Zucker diabetic fatty (ZDF) rats constitute a well-documented model for T2DM because these animals have a mutation that is responsible for leptin receptor deficiency and develop insulin-resistant hyperglycaemia and metabolic syndrome at the age of 9 weeks [17]. The ZDF model was used to demonstrate a significant association between T2DM and decreased BMD, trabecular bone volume (Tb.BV), and cortical thickness (Ct.Th) in the vertebrae and in the distal metaphyses of femurs [18–21]. The ZDF model was also used to test several hypotheses, such as an increase in non-enzymatic glycation [15] [22–24] and reduction in bone turnover [25–27], regarding the impaired bone quality observed in T2DM. However, to the best of our knowledge, there is no data available in the literature on the proximal femur, which is a determinant location for the fragility fractures, or on cortical porosity, which is a determinant factor for bone strength. Finally, although microangiopathy is involved in most T2DM complications, bone vascularisation and its potential impaction with bone microstructure remains poorly documented.

To better understand bone fragility in T2DM and define the contribution of microcomputed tomography (micro-CT) to the evaluation of bone microarchitecture and vascularisation, we conducted an *in vitro* preliminary study on ZDF and control rats. We first analysed trabecular and cortical bone microarchitecture of the proximal, mid-diaphyseal, and distal femur, then determined whether micro-CT allowed to explore bone vascularisation of the femurs, and finally looked for a link between microstructure and vascularisation parameters.

## Methods

### Animal model

The experimental procedures involving the rats used in the present study were approved by the Ethics Committee of the University Paris Diderot (No. 01610.01/S69), Paris, France. Twenty-six rats were studied: 14 ZDF rats and 12 age-matched Zucker lean (ZL) rats as controls. Among them, 8 ZDF and 6 ZL rats were investigated for bone microarchitecture (group 1), while 6 ZDF and 6 ZL rats were studied for bone vascularisation (group 2). The animals were maintained under standard animal laboratory conditions with a light-dark cycle of 12 h and a temperature of 20–21 °C. The animals had free access to water and were fed with high fat and high carbohydrate foods. At 21 weeks, blood sugar level and alkaline phosphate, fructosamine, and calcium blood level were determined, and the animal body weight was measured. At 24 weeks, the rats of group 2 (6 ZDF and 6 ZL rats) received an injection of Microfil® (Microfil red compound MV 130, Flow Tech Inc., Carver, MA, USA) which is a radiopaque casting agent. At that time, the 26 rats were euthanised and their right femur excised for analyses.

### Image acquisition

Micro-CT images were acquired using a Skyscan 1172 micro-CT (Bruker, Hambourg, Germany), at three sites of the excised femurs: proximal and distal ends, and shaft, of the femur of both ZDF rats (*n* = 8) and ZL rats (*n* = 6). The micro-CT images were acquired at the following setting: 80 kVp, 100 mAs, matrix 2000 × 1336, exposure time 110 ms, filter Al 0.5, and voxel size 6 μm.

### Image analysis

Image analysis was performed using the CTAn software (Bruker, Hambourg, Germany).

#### Definition of the volume of interest (VOI) for trabecular bone and cortical bone

Trabecular bone was analysed in the head, neck, and distal metaphysis of the femurs. Cortical bone was analysed in the neck and shaft. VOIs were determined using semi-automatic and robust segmentation techniques. We obtained the VOIs from several selected images and the software then interpolated between them. Details regarding image selection for each VOI were as follows:

- For the *head*, the first axial image in the head served as a reference; we moved forward by 100 images and plotted the VOI on the following 300 images.
- For the *neck*, images were first reoriented along the direction of the neck axis and the first image in the neck was chosen as a reference; we moved forward by 100 images for trabecular bone and 200 images for cortical bone; the VOI extended over 100 images for cortical and trabecular bone.
- For the *shaft,* the reference section was the growth plate; we moved forward by 500 images and plotted the VOI between the endosteum and periosteum on 100 images.
- For the *distal metaphysis*, the growth plate served as a reference; we moved forward by 300 images to plot the VOI within the endosteum; the VOI extended over 300 images.

#### Thresholding

Trabecular and cortical bone were investigated using global thresholding. For each trabecular bone specimen, the threshold was fixed at the minimal intensity between the two curves of the histograms representing bone and bone marrow. This threshold allowed for separation of the bone from the bone marrow. Due to the small pore surface area and partial volume effects in cortical bone, only one curve representing bone tissue was obtained. For this reason, the threshold for each specimen was placed on the minimum intensity of the bone tissue, before the beginning of the curve. This threshold allowed us to separate the pores from the bone tissue.

#### Assessed variables

The trabecular bone variables assessed were as follows: trabecular bone volume (Tb.BV), trabecular bone percentage (Tb.BV/TV), trabecular thickness (Tb.Th), trabecular number (Tb.N), trabecular separation (Tb.Sp), index of connectivity (Conn.D), structural model index (SMI), and volumetric bone mineral density (vBMD). The cortical bone variables assessed were as follows: cortical bone percentage (Ct.BV/TV), cortical surface (Ct.Ar), cortical perimeter (Ct.P), cortical thickness (Ct.Th), cortical porosity (Ct.Po), and volumetric bone mineral density (vBMD).

### Microfil® vascular injection and micro-CT parameters of bone vasculature

Bone vascularisation was determined using 12 rats (6 ZDF rats and 6 ZL rats). In order to obtain three-dimensional views of the rat vascular network using micro-CT, Microfil® (MicrofilTM red compound MV 130, Flow Tech Inc., Carver, MA, USA) [28] was injected.

The procedure started with anaesthetising the rats using 100 μL of ketamine (100 mg/mL; Pfizer, NYC, USA) and 50 μL of Xyzalin (20 mg/mL, Bayer, Leverkusen, Germany). The skin from the thorax and the rib cage of each animal was incised to access the heart, and 0.3 mL heparin (5.000 UI/mL) was injected directly in the left ventricle. After 10 min, the left ventricle was catheterised using a 22G canula and the right atrium was cut to remove the contained blood. First, animals were perfused with isotonic saline (200 mL) containing heparin (100 UI/mL) to rinse the blood vessels. Animals were then perfused via the intracardiac canula with 50 mL of Microfil® (prepared according to the manufacturer’s instructions using 20 mL of Microfil®, 25 mL of the specific diluent, and 5 mL of the specific curing agent). The pressure during filling was of approximately 120 mmHg in order to force Microfil® into the capillary networks without extravasation into the surrounding tissue. This injection was considered successful when perfect opacification of the digestive tract capillaries was observed. Such a result was obtained only in seven of the 12 rats tested (4 ZDF and 3 ZL rats). The five rats with imperfect opacification were used to determine the decalcification and micro-CT protocols. Due to the similar micro-CT values between opacified vessels and bone, the excised femurs had to be decalcified before micro-CT analysis in order to assess the presence of vessels [29]. The parameters used for Micro-CT image acquisition were 80 kVp, 100 mAs, matrix 4.000 × 2.272, time exposure 280 ms, no filters, and voxel size 3 μm.

The VOIs for the vessel analyses were identical to those defined for bone microarchitecture evaluation. A global thresholding technique was used for segmentation. For each specimen, the threshold was placed at the end of the curve that separates bone marrow and blood vessels. The following data were obtained using CTAn (Bruker, Hambourg, Germany): vessel volume (VV), vessel percentage (VV/TV %), vessel thickness (V.Th), vessel number (V.N), and vessel separation (V.Sp).

### Statistical analysis

All experimental data were compiled as means and standard deviations (mean ± standard deviation) and reported separately for each group of rats. Comparisons of the variables of bone microarchitecture and bone vascularisation between ZDF and ZL rats were carried out using a non-parametric test (Mann-Whitney *U*). All tests were two-sided with a significance level equal to 0.05 and analysis were made using SPPS for Windows (v.10, SPSS Inc., Chicago, IL, USA).

## Results

The 24-week-old ZDF and ZL rats had an average weight of 439.4 ± 16.35 g and 387.2 ± 10.35 g, respectively. The ZDF rats had higher blood serum glucose levels than the ZL rats (5.88 ± 0.17 versus 2.33 ± 0.15 (*p* < 0.001)). In addition, the ZDF rats also had significantly increased levels of fructosamine (*p* = 0.001), alkaline phosphatase (*p* = 0.005), and calcium (*p* = 0.001).

### Bone microarchitecture in ZDF and ZL rats

**Fig. 1:**
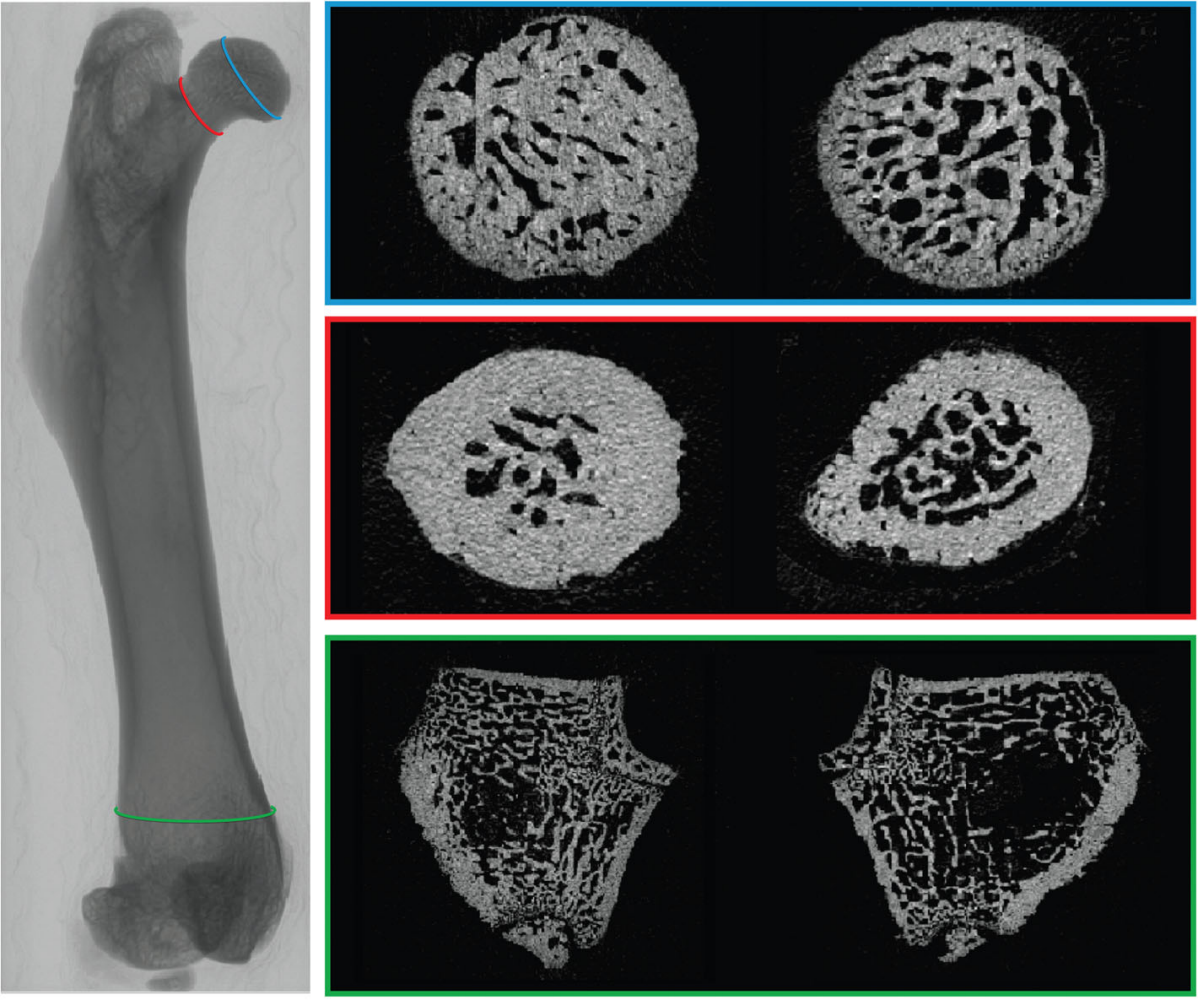
Comparison of trabecular bone between a Zucker lean rat (ZL, left) and a Zucker diabetic fatty rat (ZDF, right). Axial images of the femoral head, neck, and distal metaphysis. Decreased bone volume and trabecular number in ZDF rats compared to ZL rats, in the three volumes of interest

**Fig. 2:**
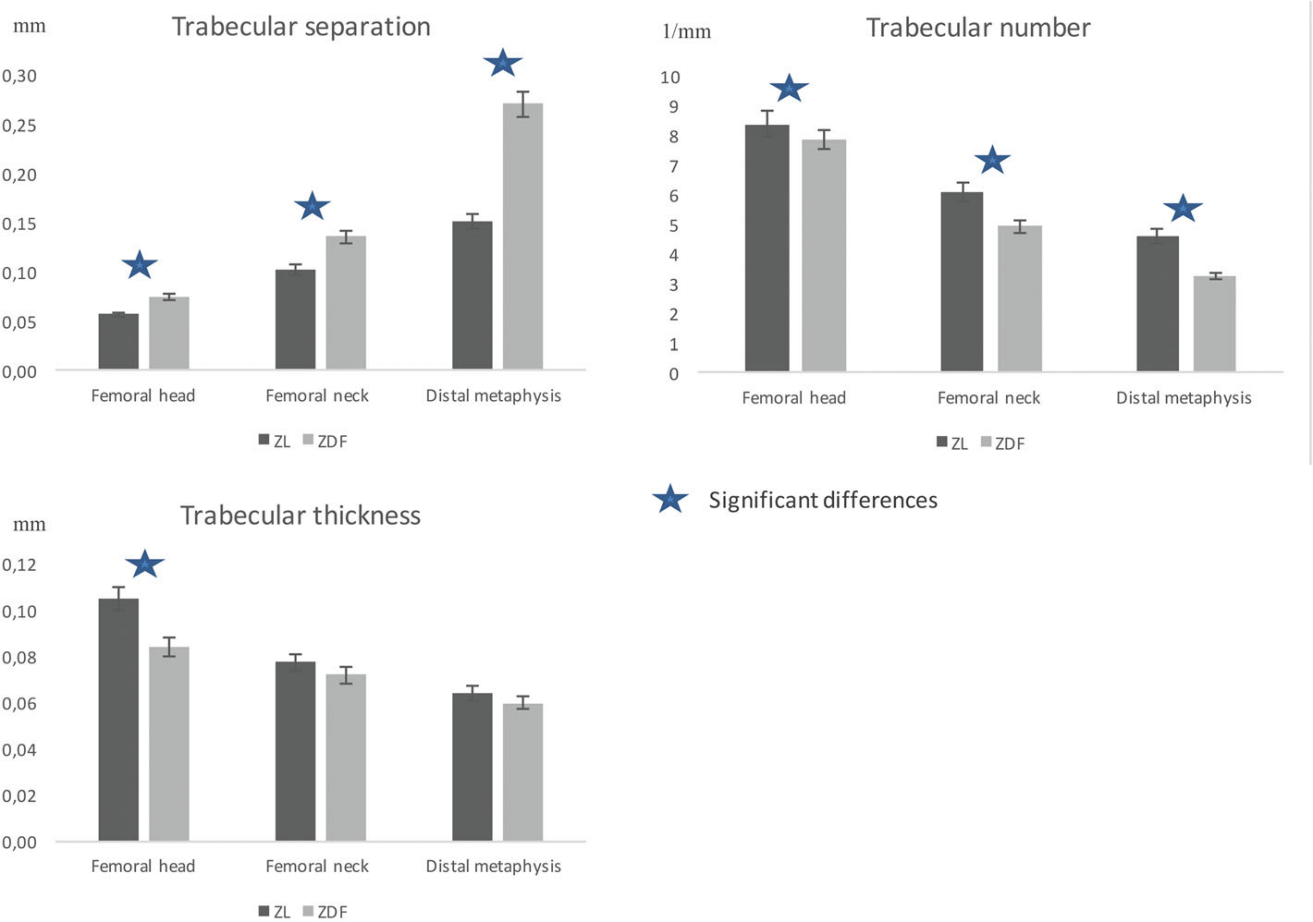
Trabecular bone characteristics of Zucker diabetic fatty (ZDF) and Zucker lean (ZL) rats. There were significant differences between ZDF and ZL rats concerning the number and the separation of trabeculae in the head, neck, and distal metaphysis. Trabecular thickness was significantly different only in the head

The results and comparisons of trabecular bone microarchitecture between ZDF and ZL rats are shown in Table 1. Compared to ZL rats, the trabecular bone of ZDF rats was significantly different. The ZDF rats had significantly lower Tb.BV values in the head (*p* = 0.003), neck (*p* = 0.02), and distal metaphysis (*p* = 0.001) (Fig. 1). Tb.N was significantly lower, and Tb.Sp significantly higher in the head (*p* = 0.02), neck (*p* = 0.02), and distal metaphysis (*p* = 0.003) of the ZDF rats. Tb.Th was significantly lower in the ZDF rats compared to the respective values obtained from the heads of ZL rats (*p* = 0.005), but this was not significant in the neck nor in the distal metaphysis (Fig. 2). SMI was significantly lower in the head, neck, and distal metaphysis of the ZDF rats (*p* = 0.02; *p* = 0.04; *p* = 0.008). Conn.D was not significantly different in the two groups. Trabecular vBMD was significantly lower in the ZDF rats only in the distal metaphysis (*p* = 0.003).

**Table 1:** Analysis of trabecular bone

	ZL rats	ZDF rats	*p*
Femoral head			
Tb.BV/TV (%)	81.01 ± 1.7	69.35 ± 4.73	0.003
SMI	- 9.74 ± 2.4	- 5.49 ± 1.6	0.02
Tb.N (1/mm)	8.38 ± 0.86	7.87 ± 1.078	0.04
Tb.Sp (mm)	0.056 ± 0.006	0.07 ± 0.014	0.02
Tb.Th (mm)	0.10 ± 0.017	0.08 ± 0.013	0.005
Conn.D (1/mm^3^)	1270 ± 197	2237 ± 646	0.07
Femoral neck			
Tb.BV/TV (%)	46.9 ± 4.61	35.37 ± 6.67	0.02
SMI	0.89 ± 0.48	1.26 ± 0.22	0.04
Tb.N (1/mm)	6.07 ± 0.62	4.90 ± 0.92	0.02
Tb.Sp (mm)	0.10 ± 0.02	0.13 ± 0.03	0.02
Tb.Th (mm)	0.077 ± 0.008	0.07 ± 0.004	0.22
Conn.D (1/mm^3^)	1320 ± 458	1312 ± 675	0.34
vBMD	0.57 ± 11	0.49 ± 0.06	0.14
Distal metaphysis			
Tb.BV/TV (%)	29.56 ± 4.61	19.38 ± 4.07	0.001
SMI	0.81 ± 0.21	1.18 ± 0.18	0.008
Tb.N (1/mm)	4.60 ± 0.62	3.25 ± 0.73	0.005
Tb.SP (mm)	0.15 ± 0.03	0.26 ± 0.05	0.003
Tb.Th (mm)	0.06 ± 0.004	0.05 ± 0.003	0.08
Conn.D (1/mm^3^)	1176 ± 348	747 ± 437	0.05
vBMD	0.39 ± 0.04	0.27 ± 0.05	0.003

Data are mean ± standard deviation. *Conn.D* index of connectivity, *Tb.BV/TV* trabecular bone volume percentage, *Tb.N* trabecular number, *Tb.Sp* trabecular separation, *Tb.Th* trabecular thickness, *vBMD* volumetric bone mineral density, *ZDF* Zucker diabetic fatty, *ZL* Zucker lean

**Fig. 3:**
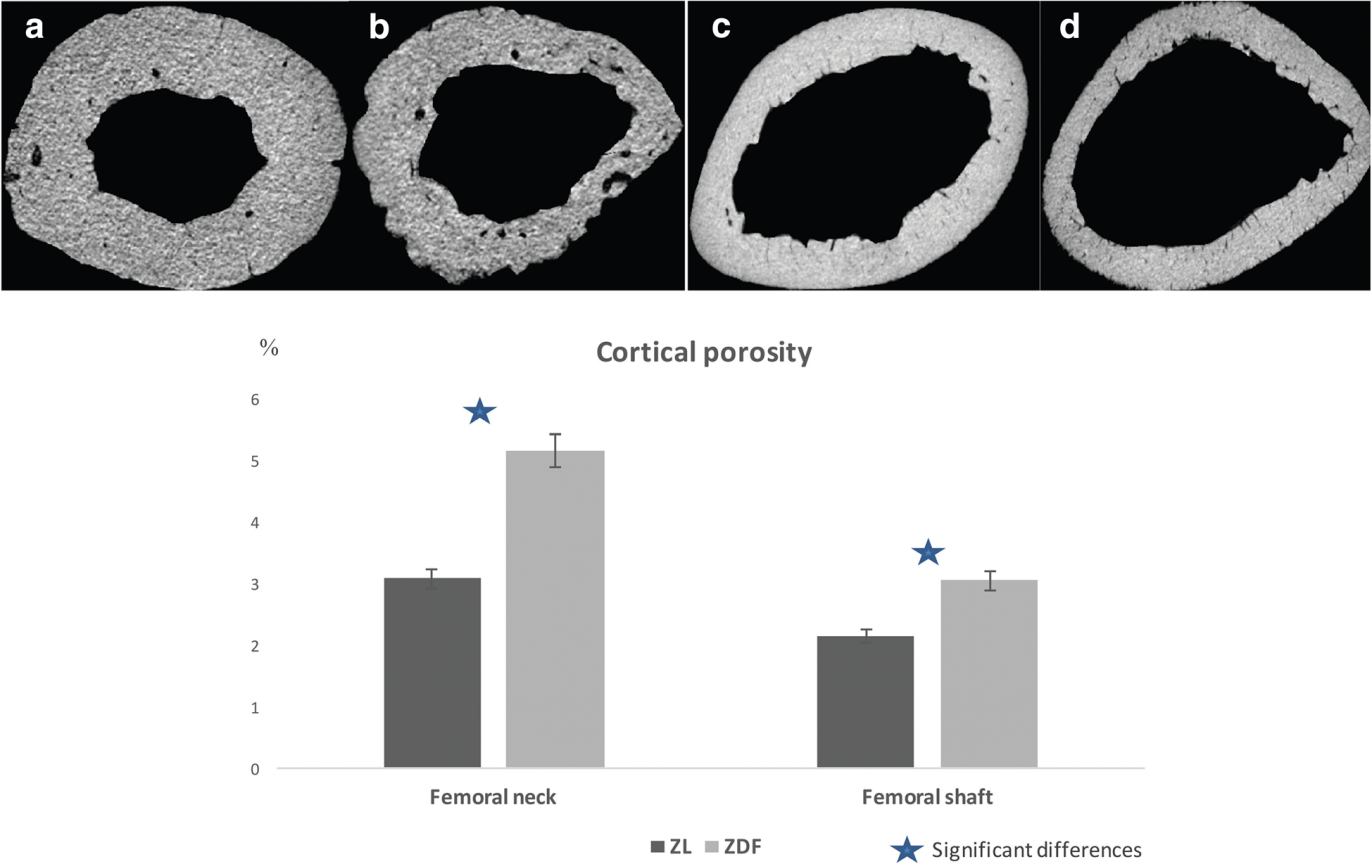
Comparison of cortical porosity between a Zucker diabetic fatty (ZDF) rat and a Zucker lean ZL rat. Axial images of the femoral neck of ZL (**a**) and ZDF (**b**) rats, and the femoral shaft of ZL (**c**) and ZDF (**d**) rats. Cortical porosity was higher in ZDF compared to ZL rats, in the two locations

The results and comparisons of cortical bone microarchitecture between ZDF and ZL rats are shown in Table 2. Assessment of the cortical bone in the neck and shaft of the femurs also revealed significant differences of several microarchitecture parameters between the two groups. Ct.BV/TV was lower in the ZDF rats, both in the neck (*p* = 0.001) and shaft (*p* = 0.005). Ct.Po was higher in the ZDF rats in the neck (5.15 ± 1.7 versus 3.07 ± 0.81; *p* = 0.001) and shaft (2.14 ± 0.54 versus 3.04 ± 0.99; *p* = 0.005 (Fig. 3). Ct.P, Ct.Th, and cortical vBMD were not significantly different between the two groups of rats.

**Table 2:** Analysis of cortical bone

	ZL rats	ZDF rats	*p*
Femoral neck			
Ct.BV/TV (%)	72.80 ± 2.96	62.9 ± 3.2	0.001
Ct.Ar (mm^2^)	2.50 ± 0.27	2.23 ± 0.12	0.043
Ct.Ar/Tt.Ar (%)	0.73 ± 0.02	0.62 ± 0.03	0.001
Ct.P (mm)	32.51 ± 5.94	31.75 ± 2.67	0.414
Ct.Th (mm)	0.15 ± 0.03	0.14 ± 0.008	0.108
Ct.Po (%)	3.07 ± 0.81	5.15 ± 1.77	0.001
vBMD	1.21 ± 0.07	1.23 ± 0.06	0.181
Femoral shaft			
Ct.BV/TV (%)	43.07 ± 1.55	40.09 ± 2.17	0.005
Ct.Ar (mm^2^)	5.49 ± 0.51	4.82 ± 0.21	0.005
Ct.Ar/Tt.Ar (%)	0.43 ± 0.01	0.40 ± 0.02	0.005
Ct.P (mm)	45.87 ± 8.13	38.71 ± 2.69	0.181
Ct.Th (mm)	0.24 ± 0.045	0.25 ± 0.025	0.950
Ct.Po (%)	3.04 ± 0.99	2.14 ± 0.54	0.05
vBMD	1.44 ± 0.137	1.39 ± 0.09	0.414

Data are mean ± standard deviation. *Ct.Ar* cortical area, *Ct.Ar/Tt.Ar* cortical area fraction, *Ct.BV/TV* cortical bone volume percentage, *Ct.P* cortical perimeter, *Ct.Po* cortical porosity, *Ct.Th* cortical thickness, *vBMD* volumetric bone mineral density, *ZDF* Zucker diabetic fatty, *ZL* Zucker lean

### Bone vasculature parameters in ZDF and ZL rats

**Fig. 4:**
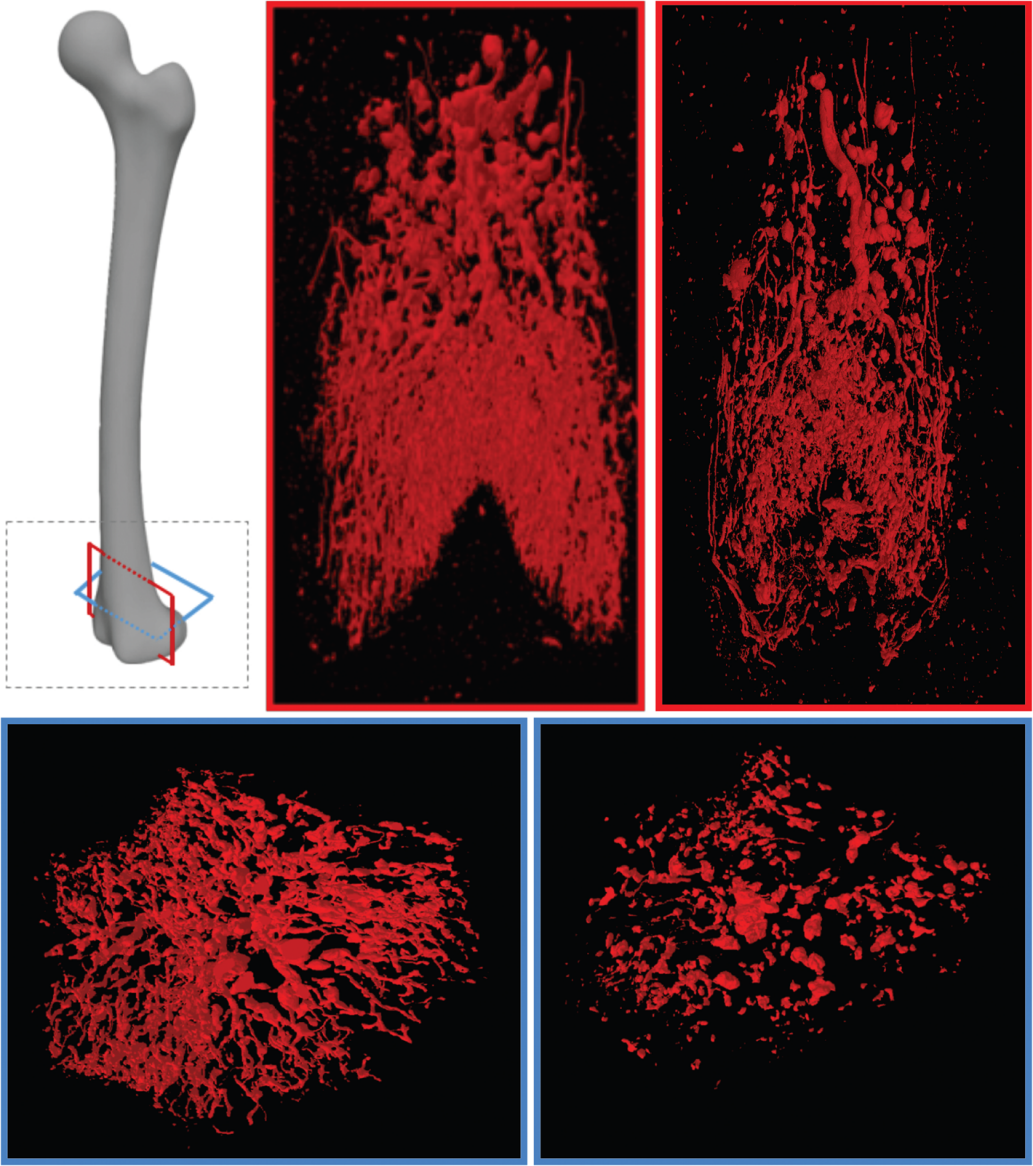
Vascularisation of distal metaphysis of Zucker lean (ZL) rats and Zucker diabetic fatty (ZDF) rats, coronal and axial three-dimensional view. There was a lower vascular volume and number in ZDF rats as compared to ZL rats

The results and comparisons of vascularisation parameters between ZDF and ZL rats are shown in Table 3. Compared to results from the ZL rats, the V.V was significantly (*p* < 0.05) lower in the ZDF rats in the neck (0.012 ± 0.02 versus 0.001 ± 0.008) and in the distal metaphysis (0.40 ± 0.12 versus 1.12 ± 0.32). There were also significantly (*p* = 0.047) lower VV/TV in the neck and distal metaphysis (*p* = 0.047) of ZDF rats compared to results obtained from the ZL rats (Fig. 4). The V.Nb values in the neck and in the distal metaphysis of the ZDF versus the ZL rats were significantly lower (0.30 ± 0.06 versus 0.64 ± 0.07, respectively, *p* = 0.047 and 0.19 ± 0.06 versus 0.53 ± 014, respectively, *p* = 0.047). V.Th values were not significantly different between the two groups.

**Table 3:** Analysis of vascularisation

	ZL rats	ZDF rats	*p*
Femoral neck			
VV	0.02 ± 0.008	0.012 ± 0.002	0.047
VV/TV (%)	2.8 ± 0.55	1.81 ± 0.21	0.047
V.N (1/mm)	0.64 ± 0.07	0.32 ± 0.06	0.047
V.Sp (mm)	0.25 ± 0.02	0.30 ± 0.01	0.047
V.Th (mm)	0.04 ± 0.002	0.04 ± 0.006	0.069
Distal metaphysis			
VV	1.12 ± 0.3	0.44 ± 0.11	0.047
VV/TV (%)	4.35 ± 1	1.8 ± 0.50	0.047
V.N (1/mm)	0.53 ± 0.14	0.21 ± 0.06	0.047
V.Sp (mm)	0.40 ± 0.018	0.64 ± 0.23	0.047
V.Th (mm)	0.30 ± 0.40	0.08 ± 0.009	0.089

Data are mean ± standard deviation. *V.N* vessel number, *V.Sp* vessel separation, *V.Th* vessel thickness, *VV* vessel volume, *VV/TV* vessel volume percentage, *ZDF* Zucker diabetic fatty, *ZL* Zucker lean

## Discussion

The objective of our study was to use micro-CT to compare trabecular and cortical bone microarchitecture and bone vasculature of the femur of ZDF 24-week rats as a model of long standing diabetic conditions to that of their ZL littermates as controls. The ZDF rats are an established model of T2DM which has been used to examine musculoskeletal complications such as bone fragility [18]. Micro-CT enables a separate evaluation of the cortical and trabecular bone compartments; it has also demonstrated its usefulness in the study of bone vascularisation in healthy rats [30–33].

We observed a significantly lower vBMD in the trabecular compartment of the distal metaphysis of the ZDF rat femurs. Other studies using ZDF rats [20, 21] reported that vBMD varies non-linearly with respect to anatomical location and that the distal femoral metaphysis was the region with the largest vBMD reduction. vBMD, as measured by micro-CT, is an *apparent mineral density*; it reflects both the amount of bone (bone volume fraction) and the degree of bone mineralisation. Since the degree of bone tissue mineralisation changes very little, vBMD changes mainly reflect changes in the bone volume fraction. Our significantly lower vBMD in the distal diaphysis was clearly related to a significantly lower trabecular bone volume and trabecular number.

Indeed, our study showed a significantly lower trabecular bone volume in the femurs of ZDF rats compared with those of ZL rats; this was observed in the three VOIs located into the head, neck, and distal metaphysis. This finding may be related to the significant differences observed in trabecular number (lower) and spacing (higher) in the ZDF rats compared to the ZL rats. The trabecular thickness was significantly lower only in the femur head of ZDF compared with ZL rats.

The observed lower trabecular bone volume is consistent with the results in literature reports of studies on the vertebrae [19, 20] and distal metaphysis of the femur [18]. In those studies, the differences observed in trabecular bone volume were explained by the number and thickness of trabeculae [17, 18] or thickness only [19]. These studies were carried out at resolutions of 12 and 20 μm on ZDF and ZL 9- and 33-week-old rats. Our study used 6 μm of resolution and 24-week-old rats. Better resolution should improve the determination of any existing differences in trabecular thickness between ZDF and ZL rats. Other studies which used ZDF rats of various ages may be needed in order to determine whether or not there is a difference.

Our differences in SMI in ZDF rats that reflect changes in the microarchitecture of trabecular bone, and may be responsible for fragility and increased fracture risk, are consistent with reports in the literature [17–19]. The papers in the literature also reported an increase in the connectivity index, indicating an anarchic reorganisation of the bony trabeculae, a result which was not observed in our study.

There is a major need to non-invasively monitor and evaluate the status of cortical bone, which plays a critical role in bone strength [14]. In this respect, mechanical testing of excised femoral necks from human cadavers has showed that the cortex contributes from 40 to 60% of the overall femoral strength [32]. Also, finite element modeling has suggested that, in the human femoral neck region, cortical bone supports 50% of the stresses associated with normal gait [33]. Moreover, administration of some newly developed antiosteoporotic drugs proved especially effective on cortical bone [34]. The mechanical properties of cortical bone depend on several structural and microstructural parameters (such as bone mineral density, cortical thickness, and cortical porosity), which can be assessed using micro-CT with appropriate spatial resolution.

Our study provided evidence of a significantly lower cortical area and of a significant increase in the cortical porosity of the neck and shaft in ZDF rats. These results are in agreement with those reported for the human femoral shaft in one study [19]. However, to the best of our knowledge, cortical porosity has never been studied in ZDF rats before.

Investigation of the bone microvasculature using micro-CT is challenging because vascular opacification is required for the three-dimensional study of the vascular tree [35]. Our study used information from published studies on the topic [28–30] to establish a protocol involving the use of Microfil® as a contrast agent. Our procedure was complex. We judged injections as satisfactory in only 7 rats out of 12. Such outcomes are consistent with the few reported studies that also obtained unsatisfactory opacification of small blood vessels [29]. Obtaining satisfactory Microfil® injections was not the only challenge. Indeed, the CT densities of bone and opacified vessels are very similar for the two tissues and cannot be distinguished on histograms. Consequently, decalcification of the bone is mandatory. Other options were discussed in the literature. Micro-CT using synchrotron radiation offers both higher spatial resolution and higher contrast and thus provides differentiation of bone tissue from blood vessels without specimen decalcification [28] but is of limited availability. Some studies reported the use of barium sulphate for opacification of blood vessels and, thus, differentiation from bone tissue [31, 33]. Analysis of periosteal vascularisation without decalcification, using a segmentation technique, has also been reported [36] but was not appropriate for our study. Our study demonstrated that bone vascularisation in the ZDF rats was poor compared to that of the ZL rats. The number and percentage of blood vessels in the neck and distal metaphysis of the ZDF rat femurs was lower. Two femoral heads among the ZDF rats tested were unfortunately broken during manipulation, preventing measurements of the vascularisation parameters in the region of interest; in fact, the vascularisation in ZDF rats was poorer than that observed in the ZL rats. The observed difference in the vascular tissue volume may be explained by the significant difference in the number of blood vessels.

To our knowledge, there is no study regarding the bone vascular network and its correlation with alterations in bone quality in the long bones of T2DM rats. Our study applied micro-CT analysis to study bone vasculature and showed impaired bone vascularisation in ZDF rats. It should be noted that the observed vascular changes were more pronounced at the distal metaphysis of the femur where major bone quantity and quality changes were observed. The underlying mechanisms for these events remain unclear. Several hypotheses such as the role of vascularisation in bone remodelling units through complex molecular communications and recruitment of bone cells from pericytes [30], or vasomotor alterations of the principal nutrient artery of the femur from ZDF rats [37], have been proposed. It is therefore possible that the microangiopathy may be at least in part responsible for the bone microarchitecture changes observed in diabetic subjects.

The present study has limitations. First, the sample size dedicated to the study of bone vessels in the femur of rats using micro-CT was quite small. Indeed, we obtained satisfactory opacification of the vessels in only seven out of 12 rats. Second, our analysis was conducted using a resolution of 6 μm, so that an accurate identification of pores was difficult as regards to pore size which varies between 5 and 0.1 mm. The use of a better resolution would probably allow for more accurate analysis of the porosity of bone in ZDF rats. Anyway, the results observed in our study are consistent with literature reports of an increased in porosity in men with T2DM [14, 38, 25].

In summary, our study using micro-CT provided evidence that, firstly, trabecular and cortical bone quantity and microarchitecture were altered in the femur of ZDF rats; secondly, bone vasculature was altered in the femur of ZDF rats; and, thirdly, the main region of interest for studying architecture and vasculature changes in the femur of ZDF rats was the distal metaphysis. Our preliminary study regarding bone vascularisation needs confirmation with larger populations.

## Abbreviations

Conn.D: Index of connectivity
Ct.Ar: Cortical surface
Ct.BV/TV: Cortical bone percentage
Ct.P: Cortical perimeter
Ct.Po: Cortical porosity
Ct.Th: Cortical thickness
micro-CT: Microcomputed tomography
SMI: Structural model index
T2DM: Type 2 diabetes mellitus
Tb.BV: Trabecular bone volume
Tb.BV/TV: Trabecular bone percentage
Tb.N: Trabecular number
Tb.Sp: Trabecular separation
Tb.Th: Trabecular thickness
V.N: Vessel number
V.Sp: Vessel separation
V.Th: Vessel thickness
vBMD: Volumetric bone mineral density
VOI: Volumes of interest
VV: Vessel volume
VV/TV: Vessel percentage
ZDF: Zucker diabetic fatty
ZL: Zucker lean
